# The Mechanism of Ammonia-Assimilating Bacteria Promoting the Growth of Oyster Mushrooms (*Pleurotus ostreatus*)

**DOI:** 10.3390/jof11020130

**Published:** 2025-02-09

**Authors:** Rui Li, Qi Zhang, Yuannan Chen, Yuqian Gao, Yanqing Yang, Qin Liu, Weili Kong, Haopeng Chai, Bingke Sun, Yanan Li, Liyou Qiu

**Affiliations:** 1 Key Laboratory of Enzyme Engineering of Agricultural Microbiology, Ministry of Agriculture and Rural Affairs, College of Life Sciences, Henan Agricultural University, Zhengzhou 450046, China; 2 Key Laboratory of Evaluation and Utilization of Germplasm Resources of Edible Fungi in Huang-Huai-Hai Region, Institute of Edible Fungi, Henan Academy of Agricultural Sciences, Ministry of Agriculture and Rural Affairs, Zhengzhou 450002, China

**Keywords:** ammonia-assimilating bacteria, *Pleurotus ostreatus*, ROS, mushroom growth promoters

## Abstract

Oyster mushrooms (*Pleurotus ostreatus*) are one of the most commonly grown edible mushrooms using compost, which contains high concentrations of ammonia. In this study, inoculation of the oyster mushroom culture substrate with ammonia-assimilating bacterium *Enterobacter* sp. B12, either before or after composting, reduced the ammonia nitrogen content, increased the total nitrogen content of the compost, and enhanced the mushroom yield. Co-cultivation with *P. ostreatus* mycelia on potato dextrose agar (PDA) plates containing 200 mM NH_4_^+^, B12 reduced reactive oxygen species (ROS) accumulation in the mycelia and downregulated the expression of the ROS-generating enzymes NADPH oxidase A (NOXA) and the stress hormone ethylene synthase 1-aminocyclopropane-1-carboxylate oxidase (ACO). It also downregulated the expression of the ammonia-assimilating related genes in the mycelia, such as glutamate dehydrogenase (GDH), glutamate synthase (GOGAT), glutamine synthetase (GS), ammonia transporter protein (AMT), and amino acid transporter protein (AAT), while upregulating its own ammonia-assimilation genes. These findings suggest that the mechanism by which B12 promoted oyster mushroom growth was that B12 assimilated ammonia, alleviated ammonia stress, mitigated ROS accumulation in the mycelia, and supplied ammonia and amino acids to the mycelia. To our knowledge, ammonia-assimilating bacteria are a novel type of mushroom growth promoter (MGP).

## 1. Introduction

Edible mushrooms are a valuable source of food and medicine. Global mushroom production has increased more than fivefold since 2000 and currently stands at 44 million tons [1]. The largest contributor was *Lentinula edodes* (shiitake mushroom) at 26%, followed by *Pleurotus ostreatus* (oyster mushroom) (21%), *Auricularia* species (black ear mushroom) (21%), *Agaricus bisporus* (button mushroom) (11%), *Flammulina velutipes* (7%), *Volvariella volvacea* (paddy straw mushroom) (1%), and other mushrooms (13%) [2]. The cultivation of edible mushrooms using agricultural and agro-industrial residues not only contributes significantly to the recycling of environmental wastes [3], but also generates numerous employment opportunities [4].

Mushroom life is closely related to other microorganisms, many of which are able to promote the growth of mushrooms, and referred to as mushroom growth-promoting microorganisms or mushroom growth promoters (MGP) [5]. Certain bacteria in the soil, compost, pasteurized substrate, and casing provide nutrients and a favorable environment for mushroom growth. The phylum Bacteroidetes in the soil provides available carbon sources for morels by degrading cellulose and chitin [6]. The spore formers [7], *Bradirhizobium* spp. [8], and other bacterial strains [9] isolated from truffles or truffle ectomycorrhizas promote the growth of the truffle mycelia and probably the establishment of symbiotic collaborations. The dominant thermophilic fungus *Mycothermus thermophilus* (previously *Scytalidium thermophilum* or *Torula thermophila*) [10] in compost produces dozens of lignocellulose-degrading enzymes [11] that degrade lignocellulose to provide nutrients for button mushrooms. The predominant Bacillales bacteria during compost conditioning may contribute to the removal of free ammonia in the compost [12] and eliminate the negative impact on button mushroom mycelial growth [13].

Many bacterial strains are selected as potent MGP for inoculation [14]. A fungus-derived *Rhizobium* strain promotes the mycelial growth of *Polyporus umbellatus* and *Armillaria gallica* [15]. Inoculation of *Pseudomonas putida* strains and other bacterial inoculation in the casing soil increases button mushroom yield by 12–215% [16,17,18]. Inoculation of *Glutamicibacter arilaitensis* MRC119 promotes the mycelial growth and yield of oyster mushrooms [19]. However, the growth-promoting mechanism of the MGPBs remains unclear. The inoculation of *Pseudomonas* sp. UW4 in casing soil induces primordial formation and enhances the button mushroom yields. The mechanism is that UW4 produces 1-aminocyclopropane-1-carboxylic acid (ACC) deaminase, which cleaves ACC produced by the mushroom mycelia, reduces ethylene synthesis, and relieves the inhibitory effect of ethylene on the primordial formation [20,21]. The indole acetic acid (IAA) producer *Pseudomonas* sp. P7014 promotes the mycelial growth of *Pleurotus eryngii* [22].

The other function of MGP is as a biocontrol agent. Inoculation of the substrate with *Bacillus velezensis* QST713 reduces the abundance of the pathogen *Trichoderma aggressivum* in button mushroom compost [23]. Inoculation of the compost and casing soil with *Streptomyces flavovirens* A06 [24], *Pseudomonas* spp., and *Bacillus subtilis* [25] controls compost green mold disease and button mushroom brown blotch disease. However, ammonia-assimilating bacteria have not yet been characterized as MGP inoculation.

Ammonia-assimilating bacteria, which utilize ammonia by assimilation rather than nitrification [26], are prevalent in ammonia-rich environments such as the rumen [27] and compost [28,29]. These bacteria assimilate ammonia common to other organisms through the glutamate dehydrogenase (GDH) pathway and the glutamine synthetase (GS)/glutamate synthase (GOGAT) pathway [13]. Oyster mushroom compost undergoes a short composting process, and the ammonia content in the compost was higher than 0.24% [29]. In a previous study, the ammonia assimilation characteristics of the ammonia-assimilating bacterium *Enterobacter* sp. B12, isolated from the compost of oyster mushrooms, were identified. Its ammonia assimilation-related genes include the GDH gene (*gdhA*), the GS gene (*glnA*), the GOGAT gene (*gltD*), and the ammonia transporter protein gene (*amtB*). As a new type of plant growth-promoting rhizobacteria (PGPR), *Enterobacter* sp. B12 promotes plant growth by assimilating ammonia, reducing nitrogen loss, and providing ammonia and amino acids to the plant, increasing plant antioxidant capacity and ammonia tolerance [13]. Oyster mushrooms are one of the most widely cultivated edible mushrooms and are often grown on compost. However, the high concentration of ammonia in compost inhibits the mushrooms’ growth. This study aims to investigate whether B12 promotes the growth of oyster mushrooms on compost and to elucidate the mechanism of growth promotion.

## 2. Materials and Methods

### 2.1. Strains

*Pleurotus ostreatus Po*164 was provided by the Edible Fungi Germplasm Bank of the Henan Academy of Agricultural Sciences and was maintained and incubated on potato dextrose agar (PDA) medium. *Enterobacter* B12, isolated from oyster mushroom compost, was maintained and incubated on an ammonia–nitrogen medium [13].

### 2.2. Preparation of Ammonia Assimilation Bacterial Inoculum

*Enterobacter* B12 was shaking-flask cultured in a trypticasein soy broth (TSB) medium at 30 °C and 220 rpm for 24 h. The culture broth was then mixed with steam-sterilized peat and incubated at 28 °C for 5 d to prepare the inoculum. The bacterial count in the inoculum was determined by plate counting and was 2.2 × 10^8^ CFU/g.

### 2.3. Growing Oyster Mushrooms on Compost

Compost preparation and oyster mushroom culture management were performed as previously described [30]. A total of four treatments were set up: (1) control without B12 inoculum before and after composting (− −); (2) inoculation of B12 inoculum only before composting (+ −); (3) inoculation of B12 inoculum only after composting (− +); (4) inoculation of B12 inoculum before and after composting (+ +).

### 2.4. Co-Culture of Pleurotus ostreatus and Enterobacter sp. B12 on Plates

Mycelial plugs (1 cm in diameter) of *Po*164 were inoculated at the center of PDA plates containing 0 mM, 100 mM, or 200 mM NH_4_Cl, respectively. The plates were incubated at 25 °C. When the colony reached a diameter of 4 cm, 30 μL of B12 suspension (OD600 = 1.0) was inoculated at the mycelial edge. The radial growth of the mycelium was measured daily, and the growth curve was plotted. The slope of the curve during the rapid growth phase represented the mycelial growth rate.

### 2.5. Determination of Ammoniacal Nitrogen and Total Nitrogen

Ammoniacal nitrogen and total nitrogen in the fully colonized oyster mushroom mycelial compost were determined by the Kjeldahl method and flow injection analysis [31].

### 2.6. Enzyme Activity Assay

As the oyster mushroom mycelia spread across the plates, the surface mycelia from the region where both the mycelia and B12 coexisted were collected for qPCR analysis and enzyme activity assays. The activities of superoxide dismutase (SOD), peroxidase (POD), catalase (CAT), glutamine synthetase (GS), glutamate synthase (GOGAT), and glutamate dehydrogenase (GDH) were assessed using assay kits (Table 1) following the manufacturer’s instructions.

### 2.7. Mycelial Reactive Oxygen Species (ROS) Detection

ROS produced by mycelia climbing onto the coverslip was determined using the oxidant-sensitive probe dichlorodihydrofluorescein diacetate (H2DCFDA), as described elsewhere [32]. Slides were observed and imaged by using a confocal fluorescence microscope (Nikon A1HD25, Tokyo, Japan).

### 2.8. Quantitative RT-PCR

Total RNA from oyster mushroom mycelia and co-cultured *Enterobacter* sp. B12 was extracted using TRIeasy™ Total RNA Extraction Reagent (Yeasen, Shanghai, China). Total RNA was utilized as a template for quantitative RT-PCR to assess the expression of genes associated with ammonia assimilation and nitrogen metabolism, as well as the ROS-generating enzymes NADPH oxidase A (NOXA) [33] and the stress hormone ethylene synthase gene 1-aminocyclopropane-1-carboxylate oxidase (ACO) in oyster mushroom mycelia and B12. The primers are listed in Table 2. The reference genes for *P. ostreatus* and *Enterobacter* sp. B12 were *UBQ* and *rpoB*. The relative gene expression level was calculated using the 2^−ΔΔCT^ method [34].

### 2.9. Statistical Analysis

All experiments were conducted at least three times, with each assay performed in triplicate. The means from these three independent experiments were compared using one-way ANOVA followed by Tukey’s multiple-comparison test.

## 3. Results

### 3.1. Application of Enterobacter sp. B12 in the Cultivation of Oyster Mushrooms with Compost

**Figure 1:**
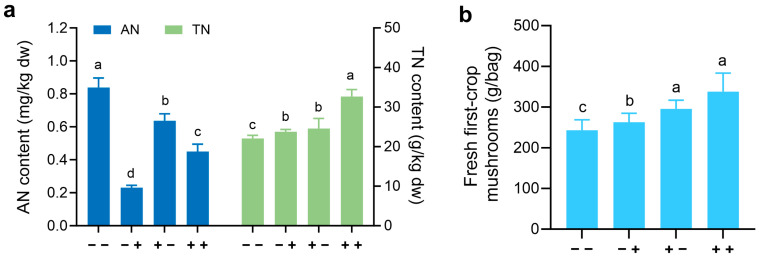
Effect of inoculation of oyster mushroom culture substrate with *Enterobacter* sp. B12 on ammonia nitrogen, total nitrogen in compost, and mushroom yield. (**a**) Ammonia nitrogen and total nitrogen content in compost. (**b**) Fresh weight of the first-crop mushrooms. − −, control without B12 inoculum before and after composting; + −, inoculation of B12 inoculum only before composting; − +, inoculation of B12 inoculum only after composting; + +, inoculation of B12 inoculum before and after composting. AN, ammonia nitrogen; TN, total nitrogen; same color bars with different lowercase letters indicated significant difference (*p* < 0.05).

Inoculation of the oyster mushroom culture substrate with *Enterobacter* sp. B12 inoculum resulted in a faster temperature rise and reduced composting time by one day compared to the control without inoculum. After composting, the inoculation of oyster mushrooms, and cultivation until the culture bags were completely colonized by mycelia, whether B12 was inoculated before or after composting, the ammonia nitrogen content in the compost decreased, the total nitrogen content increased, and the yield of first-crop mushrooms increased. In particular, inoculation of B12 before and after composting proved to be particularly effective (Figure 1).

### 3.2. Impact of Enterobacter B12 on Mycelial Growth of Pleurotus ostreatus in Ammonia-Rich Medium

**Figure 2:**
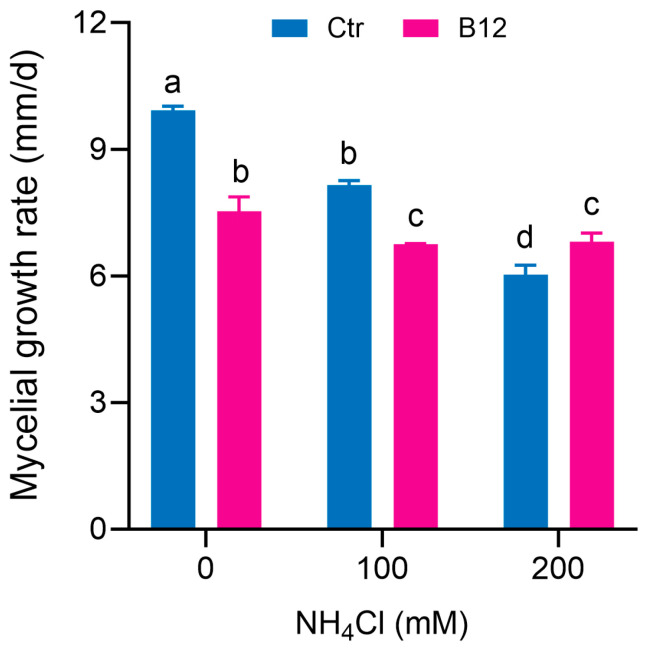
Effects of *Enterobacter* sp. B12 on the growth of *P. ostreatus* mycelia co-cultured on PDA plates with varying concentrations of ammonia. Ctr, control; B12, *Enterobacter* sp. B12. Bars marked with different lowercase letters showed a significant difference (*p* < 005).

*P. ostreatus Po*164 was grown on PDA plates containing different concentrations of ammonia; 100 mM and 200 mM ammonia inhibited the mycelial growth compared to 0 mM ammonia. When *Enterobacter* B12 was inoculated at the edge of the colony, B12 inhibited the mycelial growth when the ammonia concentration was 0 and 100 mM, which may be due to nutrient competition between B12 and the mycelia. However, when the ammonia concentration was 200 mM, B12 promoted the mycelial growth (Figure 2).

### 3.3. Effect of Enterobacter sp. B12 on the Activities of ROS-Scavenging Enzymes and Ammonia Assimilating Enzymes in the P. ostreatus Mycelia and Mycelia–B12 Co-Culture System

**Figure 3:**
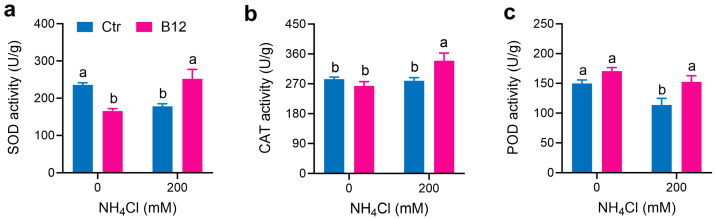
Activities of ROS-scavenging enzymes in *P. ostreatus Po*164 mycelia and mycelia–B12 co-culture system growing on PDA plates containing 0 mM and 200 mM NH_4_^+^Cl. (**a**) SOD activity. (**b**) CAT activity. (**c**) POD activity. Ctr, control; B12, *Enterobacter* sp. B12. Bars marked with different lowercase letters showed a significant difference (*p* < 005).

*P. ostreatus Po*164 was grown on PDA plates containing different concentrations of ammonia; 200 mM ammonia decreased the activities of SOD and POD in the mycelia compared to 0 mM ammonia, while CAT activity was not different. When *Enterobacter* sp. B12 was co-cultured with the mycelia at 0 mM ammonia, B12 reduced SOD activity but did not change the activities of CAT and POD in the mycelia–B12 co-culture system compared to the mycelia growing alone at 0 mM ammonia. When *Enterobacter* sp. B12 was co-cultured with the mycelia at 200 mM ammonia, B12 increased all of the SOD, CAT, and POD activities in the mycelia–B12 co-culture system compared to the mycelia growing alone at 200 mM ammonia (Figure 3).

**Figure 4:**
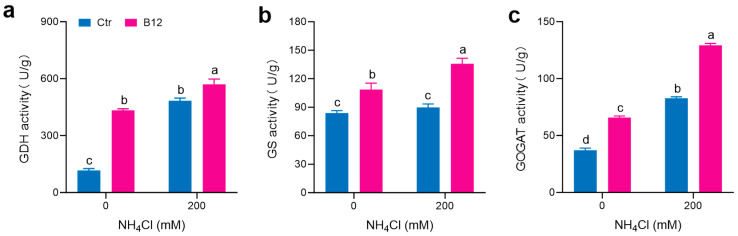
Activities of ammonia assimilating enzymes in *P. ostreatus Po*164 mycelia and the mycelia–B12 co-culture system growing on PDA plates containing 0 mM and 200 mM NH_4_Cl. (**a**) GDH activity. (**b**) GS activity. (**c**) GOGAT activity. Ctr, control; B12, *Enterobacter* sp. B12. Bars marked with different lowercase letters showed a significant difference (*p* < 005).

*Po*164 was grown on PDA plates containing different concentrations of ammonia; 200 mM ammonia decreased the activities of GDH and GOGAT in the mycelia compared to 0 mM ammonia, while GS activity was not different. When *Enterobacter* sp. B12 was co-cultured with the mycelia at 0 mM ammonia, B12 increased all of the GDH, GS, and GOGAT activities in the mycelia–B12 co-culture system compared to the mycelia growing alone at 0 mM ammonia. When *Enterobacter* sp. B12 was co-cultured with the mycelia at 200 mM ammonia, B12 increased all of GDH, GS, and GOGAT activities in the mycelia–B12 co-culture system compared to the mycelia growing alone at 200 mM ammonia (Figure 4).

### 3.4. Effect of Enterobacter sp. B12 on ROS Level in P. ostreatus Mycelia

**Figure 5:**
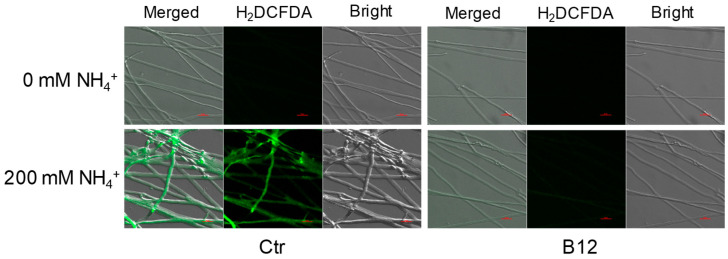
ROS levels in *P. ostreatus* mycelia induced by ammonia. Ctr, control; B12, *Enterobacter* sp. B12. Scale bars are 100 µm.

*P. ostreatus Po*164 was grown on PDA plates containing 0 mM and 200 mM NH_4_Cl with *Enterobacter* sp. B12 inoculated at the edges of the mycelial colonies. A coverslip was placed outside the bacterial inoculation circle to observe the ROS level in the climbing mycelia. In 0 mM NH_4_Cl plates with and without B12, there was no accumulation of ROS in the mycelia. In PDA plates with 200 mM NH_4_Cl, obvious ROS accumulated in the mycelia without B12, but no ROS accumulated in the mycelia with B12 (Figure 5).

### 3.5. Gene Expression in P. ostreatus Mycelia and Enterobacter sp. B12 in Co-Culture

**Figure 6:**
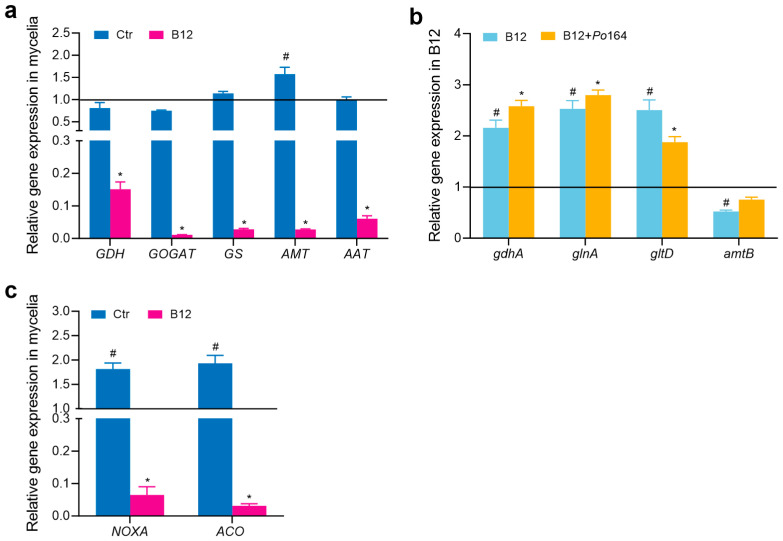
Effect of co-culture on the gene expression in *P. ostreatus* mycelia and *Enterobacter* sp. B12 on PDA plates with 200 mM NH_4_Cl. (**a**) The gene expression level in mycelia was related to the mycelia growing alone in the plates without NH_4_Cl. Ctr, control; B12, mycelia co-cultured with *Enterobacter* sp. B12. Bars marked # indicated a significant difference (*p* < 0.05) to the gene expression level in mycelia growing alone in the plates without NH_4_Cl; bars marked * showed a significant difference (*p* < 0.05) to the gene expression level in mycelia growing alone in the plates with 200 mM NH_4_Cl. (**b**) The gene expression level was related to B12 culturing on the plates without NH_4_Cl. B12, *Enterobacter* sp. B12 growing alone; B12 + *Po*164, *Enterobacter* sp. B12 co-cultured with *Po*164 mycelia. Bars marked # indicated a significant difference (*p* < 0.05) to the gene expression level in B12 growing alone on the plates without NH_4_Cl; bars marked * showed a significant difference (*p* < 005) to the gene expression level in B12 growing alone on the plates with 200 mM NH_4_Cl. (**c**) The gene expression level was related to the mycelia growing alone on the plates without NH_4_Cl. Ctr, control; B12, mycelia co-cultured with *Enterobacter* sp. B12. Bars marked # indicated a significant difference at *p* < 005 to the gene expression level in the mycelia growing alone in the plates without NH_4_Cl; bars marked * showed a significant difference at *p* < 005 to the gene expression level in the mycelia growing alone on the plates with 200 mM NH_4_Cl.

The mycelia of *P. ostreatus Po*164 was grown on PDA plates containing 200 mM NH_4_Cl; the expression of AMT, NOXA, and ACO was upregulated, while the expression of GDH, GOGAT, GS, and AAT was not changed compared to growing in 0 mM NH_4_Cl. When the mycelia co-cultured with *Enterobacter* sp. B12 at 200 mM NH_4_Cl, the expression of all the genes of GDH, GOGAT, GS, AMT, AAT, NOXA, and ACO were downregulated compared to the mycelia growing alone on the plates with 200 mM NH_4_Cl (Figure 6a,c). B12 was grown on PDA plates containing 200 mM NH_4_Cl; the expression of *gdhA*, *glnA*, and *gltD* was upregulated, but *amtB* was downregulated compared to growing in 0 mM NH_4_Cl. When co-cultured with *Po*164 mycelia, the expression of *gdhA*, *glnA*, and *gltD* was upregulated, but *amtB* was not changed compared to B12 growing alone (Figure 6b).

## 4. Discussion

Button mushrooms and oyster mushrooms are the two most widely grown edible mushrooms using compost. Composting produces a large amount of ammonia, reaching over 0.24% [28,29]. Ammonia levels in the compost at the time of spawning approaching 0.07% are inhibitory to button mushrooms [35]. Similarly, an ammonia concentration of more than 0.25% in the compost inhibits the growth of oyster mushrooms [36].

Ammonium assimilation in *A. bisporus* is mainly catalyzed by the GS/GOGAT pathway [37]. Ammonia and glutamine inhibit the activity of NADP-dependent glutamate dehydrogenase (NADP-GDH) and GOGAT [38,39], which may be the cause of ammonia intolerance in *A. bisporus*. The free ammonia produced during the composting phase I requires a conditioning process during phase II to reduce ammonia levels to approximately 0.001% in the button mushroom compost [40,41]. Ammonia assimilation in *P. ostreatus* is the only GS/GOGAT pathway [42]. High concentrations of ammonia and glutamate do not inhibit the activity of GS [43], which may be the reason why *P. ostreatus* has a higher ammonia tolerance than *A. bisporus*.

Ammonia toxicity primarily arises from non-ionic ammonium (NH_3_) rather than ionic ammonium (NH_4_^+^). NH_3_ is a fat-soluble component that can penetrate biofilms and enter cells, increasing the concentration of malondialdehyde (MDA) in the liver and decreasing the activity of superoxide dismutase (SOD), CAT, GSH-PX, and the relative expression levels of related genes [44,45]. Similarly, high ammonia stress leads to the accumulation of ROS in plant cells [46] and the release of ethylene [47]. Ammonia toxicity has not been well characterized in fungi. This study found that high ammonia inhibited the mycelial growth of *P. ostreatus* (Figure 2), reduced SOD and POD enzyme activities (Figure 3), induced ROS accumulation (Figure 5), and raised the expression of the ROS-generating enzyme gene *NOXA* and the stress hormone ethylene synthase gene *ACO* in *P. ostreatus* mycelia (Figure 6c).

In this study, the ammonia-assimilating bacterium *Enterobacter* sp. B12 accelerated the composting process, reduced the ammonia content, increased the total nitrogen content in the compost, improved the mushroom yield (Figure 1), and promoted the mycelial growth of *P. ostreatus* under high-ammonia conditions (200 mM ammonia) (Figure 2), establishing it as a novel MGP. Its growth-promoting mechanism is related to its ability to assimilate ammonia, reduce the ammonia concentration, increase the activities of ROS-scavenging enzymes (Figure 3) and ammonia-assimilating enzymes (Figure 4) in the *P. ostreatus*–B12 co-culture system, decrease the expression of *NOXA* and *ACO* genes in mycelia (Figure 6c), and mitigate ammonia stress-induced ROS accumulation in mycelia (Figure 3). The activities of ROS-scavenging enzymes and ammonia-assimilating enzymes in the *P. ostreatus*–B12 co-culture system are derived from both mycelia and B12. However, since the expression of ammonia-assimilating enzyme genes in mycelia is downregulated in the co-culture system (Figure 6a), while those in B12 are upregulated (Figure 6b), the increased activity of ammonia-assimilating enzymes in the co-culture system primarily originated from B12.

In this study, we also observed a remarkable event. When *P. ostreatus* mycelia were co-cultured with *Enterobacter* sp. B12 at high ammonia, the ammonia-assimilating enzyme activities of B12 increased (Figure 4), and its ammonia assimilation-related genes were upregulated (Figure 6b). In contrast, the ammonia assimilation genes were downregulated in the mycelia, along with the expression of AMT and AAT (Figure 6b), similar to that in the B12–wheat co-culture system [13]. This suggests the presence of ammonia and amino acid exchange between B12 and the mycelia. Such an exchange of NH_4_^+^ and NO_3_^−^ has only been reported between mycorrhizal fungi and plants [48], and possibly with amino acid exchange [49,50]. Correspondingly, inoculation with arbuscular mycorrhizal fungi downregulated the abundance of ammonium transporters ZmAMT1;1a and ZmAMT1;3, which absorb NH_4_^+^ in maize roots, upregulated ZmAMT3;1, which transports ammonium across the peri-arbuscular membrane (PAM) [51], and also downregulated the expression of the ammonium transporter genes in roots of wheat [52] and *Catalpa bungei* [53]. Thus, B12 and *P. ostreatus* formed a close symbiotic association in nitrogen metabolism.

## 5. Conclusions

High concentrations of ammonia inhibited the mycelial growth of *P. ostreatus*, decreased the activities of SOD and POD, induced the accumulation of reactive oxygen species (ROS), and increased the expression of *NOXA* and *ACO* genes. However, the ammonia-assimilating bacterium *Enterobacter* sp. B12 accelerated the composting process of oyster mushroom substrates, reduced ammonia levels while increasing total nitrogen content in the compost, increased mushroom yield, and promoted mycelial growth of *P. ostreatus* under high-ammonia conditions. Its growth-promoting mechanism is likely to involve ammonia assimilation, which reduces ammonia concentration, mitigates ROS accumulation in the mycelia caused by ammonia stress, and supplies ammonia and amino acids to the mycelia. To the best of our knowledge, ammonia-assimilating bacteria represent a novel type of MGP. Overall, inoculating the culture substrate either prior to or after composting with ammonia-assimilating bacterium is a straightforward approach to promoting oyster mushroom growth.

## Figures and Tables

**Table 1:** The kits used to measure the enzymatic activities.

Kit Name	Manufacturer	Item No.	Methodology
Superoxide Dismutase (SOD) Activity Assay Kit	Solarbio, Beijing, China	BC5165	WST-1 method
Peroxidase (POD) Activity Assay Kit	Solarbio, Beijing, China	BC0090	Spectrophotometry
Catalase (CAT) Activity Assay Kit	Solarbio, Beijing, China	BC4785	Ammonium molybdate method
Micro Glutamine Synthetase (GS) Assay Kit	Solarbio, Beijing, China	BC0915	Spectrophotometry
Micro Glutamate Synthase (GOGAT) Assay Kit	Solarbio, Beijing, China	BC0075	Spectrophotometry
Micro Glutamic Acid Dehydrogenase (GDH) Assay Kit	Solarbio, Beijing, China	BC1460	Spectrophotometry

**Table 2:** The primers used for quantitative RT-PCR.

Species	Genes	Forward Primers (5′→3′)	Reverse Primers (5′→3′)
*P. ostreatus*	*UBQ*	TCTGCTCGATGTTGACTGATC	TATTTCCTCGTCCATTCCCT
	*ACO*	GGGCAATAATGTCTGGCTCA	AGGCGTGGGATATTTCGTT
	*NOXA*	GCCGAGCGACTAGACTTTCC	GTCACCGACTTGGCGAATG
	*GOGAT*	GCTGGCGTCGGGCTTATTT	TATGGTCGGCTTTGGCTTT
	GS	CCAAGAAGGCAGGCGAATC	ATAGCCACGCCGAACTCTG
	*AAT*	GAACGCTCTACGGTCTCGC	AAGACTCGGGCACTGGATG
	*AMT*	ACCGCCTTGAGAAGAAATGG	TGCCAACGATACCTCCAACA
	*GDH*	TTGAAGGCTCCGACCTGTT	TGCGTTGACACCGTATTTGT
*Enterobacter* sp.	*glnA*	CCAACCACCAACTCCTACAAG	CGGGATACGGATAGAAGCAG
	*gltD*	AGTGACACGGGCAGCAAAT	TCGAGGCCACCCATGATAT
	*amtB*	GCAATGCGTTCTTTGGTAAC	TAGGCACATAGGAGAGCGTC
	*gdhA*	TGTGAAATCAAAGCCAGCC	CACGCCGTTGCTAATCAA
	*rpoB*	GCCAAGCCGATTTCTGGAGCA	CGTTTCGATTGGACATACG

## Data Availability

The original contributions presented in this study are included in the article. Further inquiries can be directed to the corresponding authors.
